# Use of Nonprescription and Prescription Drugs and Drug Information Sources among Breastfeeding Women in Japan: A Cross-Sectional Study

**DOI:** 10.3390/ijerph191811722

**Published:** 2022-09-17

**Authors:** Yukiko Fujii, Keiko Hirokawa, Yuko Kobuke, Toshio Kubota, Taketo Yoshitake, Koichi Haraguchi, Yukiko Honda, Hatasu Kobayashi, Kouji H. Harada

**Affiliations:** 1 Department of Pharmaceutical Science, Daiichi University of Pharmacy, Fukuoka 815-8511, Japan; 2 Sankyu-Drug Ltd., Fukuoka 801-0825, Japan; 3 Department of Community Medicine, Nagasaki University Graduate School of Biomedical Sciences, Nagasaki 852-8523, Japan; 4 Department of Environmental and Molecular Medicine, Mie University Graduate School of Medicine, Mie 514-8507, Japan; 5 Department of Health and Environmental Sciences, Kyoto University Graduate School of Medicine, Kyoto 606-8501, Japan

**Keywords:** breast feeding, nonprescription drugs, prescription drugs, medicine use, drug information, postpartum, Japan

## Abstract

Breastfeeding women may experience various health issues that require medication. This survey aimed to gain insights into the use of nonprescription and prescription drugs by breastfeeding women in Japan. A cross-sectional study involving women with children aged under two years was conducted in Fukuoka, Japan. Nonprescription drugs were used by 26% of participants in the breastfed-only group, 41% in the breastfed more than half the time group, 55% in the formula-fed more than half the time group, and 82% in the formula-fed-only group. We found that when breastfeeding rates decreased, the use of nonprescription drugs increased (*p* < 0.05, Cochran–Armitage test for trend). There were significant differences in the use of nonprescription cold medicines and oral analgesics between the formula-fed and breastfed groups, but a nonsignificant difference in prescription drugs use between the groups. These results indicated breastfeeding had a significant influence on use of nonprescription drugs, which was not observed with prescription drugs. Breastfeeding women commonly used the Internet to obtain information on both nonprescription and prescription drugs; however, this did not influence medication use.

## 1. Introduction

The World Health Organization recommends complete breastfeeding for the first six months of life and partial breastfeeding until the child is at least two years old [1]. Medical advice promoting breastfeeding is given in Japan based on this recommendation, mainly by midwives, but whether to breastfeed or use formula is left to the mother’s discretion. Breastfeeding women may experience various health issues that require medicine use. An Australian study reported that in the three months following birth, 51% of women had tiredness and exhaustion, 42% had backache, 24% had trouble losing weight, and 19% had constipation [2]. Another study from Australia showed health issues among breastfeeding women included infections such as mastitis (50%) and depressive disorders (21%) [3]. Moreover, women took more medications during breastfeeding than during pregnancy [4]. However, attention should be paid to medication use during breastfeeding and the potential effects on the child [5]. Infant nutrition is a public health issue because of the medical and neurodevelopmental advantages of breastfeeding [6], and the proper management of medicine use is important for well-being during the breastfeeding period. Previous studies examined sources of drug safety information that health professionals (e.g., medical doctors and pharmacists) used to instruct breastfeeding women [7,8,9]. However, few studies have investigated the perspectives of breastfeeding women. Therefore, little is known about where/how breastfeeding women get drug information.

There are two types of medicines in Japan: (1) prescription drugs, which are prescribed by doctors and dispensed by pharmacists at pharmacies and can only be obtained with a doctor’s prescription, with some rare exceptions, and (2) nonprescription drugs, which are able to be purchased at pharmacies without any prescription. Under the Japanese universal health insurance system, the maximum self-payment for medical expenses (including medical examination, prescriptions, and prescription drug costs) is 30% [10]. However, nonprescription medicines are not covered by the universal health insurance system and are fully self-paid, but are widely used because of their convenience. The production value of nonprescription drugs in Japan is approximately 10% of that of prescription drugs [11]. Information on prescription drugs is provided by prescribing doctors and dispensing pharmacists [12]. Information on nonprescription drugs is provided by pharmacists or sales clerks who are licensed to sell nonprescription drugs at a pharmacy and may also be obtained from drug package inserts.

When breastfeeding women experience health problems, they may visit a community pharmacy to purchase nonprescription drugs [13] or go to clinics and hospitals, which suggests that nonprescription drugs are also in demand among breastfeeding mothers. Both prescription and nonprescription drugs need to be examined to understand breastfeeding and medication status. A previous study from Japan that used the text-mining method extracted the wording “commercially available” as anxiety-related content in terms of medicine use during pregnancy [14]. This suggested that breastfeeding women may be concerned about nonprescription drugs marketed at pharmacies, as well as prescription drugs.

The present study was conducted to clarify the use of prescription and nonprescription drugs during the breastfeeding period in Japan. The specific aims were to determine the types of drugs that breastfeeding women used and compare them with those used by women who did not breastfeed or breastfed less frequently. The sources of drug information during the breastfeeding period and the influence on medicine use were also examined to gain insights to promote healthy breastfeeding and well-being.

## 2. Materials and Methods

### 2.1. Study Design, Setting and Participants

A cross-sectional study was conducted to investigate the relationship between breastfeeding and medicine use. Questionnaire items were based on a review of the literature [13,15,16,17] and expert opinion, and are listed in Table S1. We conducted a questionnaire survey in Fukuoka prefecture from February to August 2016. Convenience sampling was used to recruit participants. Participants comprised healthy women with children aged under two years who attended a childcare event at community pharmacies.

The Ethics Committee of Daiichi University of Pharmacy approved this study (no. 15013, 2 November 2015), and the study was performed in accordance with the Japan ethical guidelines for medical and health research involving human subjects.

### 2.2. Data Collection

All data were collected by the present authors and students of Daiichi University of Pharmacy. Paper-based questionnaires were distributed to eligible women at community pharmacies on seven randomly selected days in the study period. All participants were informed of the aims of this study before questionnaires were distributed. Written informed consent was obtained from all participants. If a participant’s child had already finished breastfeeding, responses relating to the period during ingestion of breast milk were obtained. Questionnaires were completed anonymously to maintain participants’ privacy.

### 2.3. Measurements

#### 2.3.1. Breastfeeding

Participants were categorized into five groups: (1) breastfed only, (2) breastfed more than half the time (breastfed for more than half of the period of ingestion of breast milk), (3) half breastfed (half breastfed and half formula fed), (4) formula fed more than half the time (formula fed for more than half of the period of ingestion of breast milk), and (5) formula fed only. In this study, the breastfeeding (BF) group in this study referred to groups (1) and (2), and the formula fed (FF) group referred to groups (4) and (5). Participants in group (3) were excluded from the analyses to avoid confusion.

#### 2.3.2. Use of Nonprescription and Prescription Drugs

Participants were asked about their medicine use during the breastfed/formula-fed period. Participants who answered “yes” were asked to select all nonprescription and prescription drugs they used from lists provided. The options for nonprescription drugs were cold medicines, medicines for headache and pain relief (oral analgesics), drugs for menstrual pain, digestive medicines, drugs for skin blotches, drugs for hay fever, motion sickness drugs, eczema drugs, vitamin preparations, anti-itching cream for insect bites/insect repellants, athlete’s foot remedies, drugs for dry skin, acne medications, medical agents for constipation, drugs for muscle aches, drugs for stiff shoulders or back pain, drugs for healthy hair, drugs for sleep, vulnerary, fomentation, other supplements, and kampo medicine. The options for prescription drugs were cold medicines, vitamins, eczema drugs, antiallergic drugs, antiasthmatics, thyroid drugs, drugs for peptic ulcer/digestive medicine, analgesic ointments/creams, anti-infection drugs, antidepressive drugs, oral contraceptives, antithrombotic agents, anxiolytics, sedatives, antipsychotics, antirheumatics, antiacne preparations, blood sugar-lowering agents, lipid-lowering agents, other cardiac drugs, antiepileptics, ovulation-inducing drugs, drugs for postpartum hemorrhage, antihypertensives, laxatives, iron preparations, oral analgesics, and kampo medicine. Nonprescription drugs are medicines that are purchased at pharmacies based on symptoms, so some item descriptions for these options differed even if they were the same type of medicines used for prescription drugs. Kampo medicine is a group of oriental medicines mainly based on herbal products. In total, 148 kampo medicines are covered by Japan’s national health insurance system [18] and are able to be purchased from a pharmacy.

#### 2.3.3. Drug Information Sources

Participants were asked to indicate their information sources for nonprescription and prescription drugs. The options for information sources were prescribing doctor, other doctors (e.g., family doctor), dispensing pharmacist, pharmacist at the pharmacy medicine was purchased, other pharmacists, sales clerks at the pharmacy of purchase, nurses/midwives, administrative agency (e.g., health center), family/friends, books, the Internet, and drug package inserts.

### 2.4. Data Analysis

Differences were examined with Pearson’s chi-square or Fisher’s exact tests if more than one of the expected values was less than 5. *p*-values < 0.05 were considered statistically significant. JMP^®^ 13 (SAS Institute Inc., Cary, NC, USA) was used for the statistical analyses.

## 3. Results and Discussion

### 3.1. Participants

Of the 142 eligible participants, 131 completed the questionnaire (92% response rate). Overall, 46 participants breastfed only, 51 breastfed more than half the time, 20 formula-fed more than half the time, and 11 formula-fed only. We excluded three participants that breastfed and formula-fed equally. Therefore, 97 participants were in the BF group, and 31 were in the FF group. There were no significant differences in the child’s age and mother’s parity between the BF and FF groups.

### 3.2. Relationship between Medicine Use and Breastfeeding

The relationship between medicine use and breastfeeding is shown in Table 1 (Q1 and Q2). The proportions of nonprescription drug use were 26% in those that breastfed only, 41% in those that breastfed more than half the time, 55% in those that formula-fed more than half the time, and 82% in those that fed formula only. This showed that as breastfeeding proportions decreased, the use of nonprescription drugs increased (*p* < 0.05, Cochran–Armitage test for trend). Conversely, the proportions of participants that took prescription drugs showed a flatter trend (61% of those that breastfed only, 55% that breastfed more than half the time, 80% that formula-fed more than half the time, and 73% that formula-fed only). This showed that prescription medication use did not significantly increase despite the proportion of breastfeeding decreasing (*p* > 0.05, Cochran–Armitage test for trend). These results indicated that breastfeeding had a significant influence on the use of nonprescription drugs. Furthermore, when the intake of nonprescription and prescription drugs in the breastfed only, breastfed more than half the time, formula-fed more than half the time, and formula-fed only groups were compared, there was only a significant difference in the breastfed only group (*p* < 0.05, Fisher’s exact test). These participants were likely to avoid nonprescription drugs, which may be explained by insufficient information sources, as discussed later.

Only one of the 31 formula feeding participants did not breastfeed because of taking medication (Table 1, Q3 and Q4). A previous study from the Netherlands showed that 11.5% of participants did not breastfeed because of taking drugs [16] (drug types, nonprescription or prescription, were not mentioned). Moreover, that study indicated only 6.4% of participants stopped using a certain drug because they breastfed, which was much lower than the proportion in this study (breastfed only: 74% for nonprescription drugs, 43% for prescription drugs; *p* < 0.05) (Table 1, Q5 and Q6). These differences may be attributed to the previous research being clinic-based, whereas this study was based on community pharmacies. Other reasons for these discrepancies may be differences in medical conditions and social and cultural preferences between the two countries.

### 3.3. Medicine Use for Nonprescription and Prescription Drugs

Medicine use for nonprescription drugs is shown in Table 2 and for prescription drugs in Table 3. These results showed that both nonprescription and prescription cold medicines were among the most frequently taken drugs in the FF group. Nonprescription cold medicine was taken by 35% of participants in the FF group, which was significantly higher than in the BF group (4%) (*p* < 0.05, Fisher’s exact test). However, prescription cold medicines were taken by 39% of participants in the FF group, and 24% in the BF group (nonsignificant, Fisher’s exact test). These results showed that the use of nonprescription and prescription cold medicines was lower in the BF group (4% and 24%, respectively) compared with that in the FF group. Oral analgesics were another commonly used medicine. Nonprescription oral analgesics were used by 26% of participants in the FF group and 7% in the BF group (*p* < 0.05, Fisher’s exact test) (Table 2), whereas prescription oral analgesics were taken by 26% in the FF group and 32% in the BF group (nonsignificant, Fisher’s exact test) (Table 3). In summary, the use of nonprescription cold medicines and oral analgesics showed significant differences between the FF and BF groups, although use of these drugs based on prescription only showed a nonsignificant difference between the two groups.

Although few components of nonprescription drugs have serious effects on infants via breast milk [19], some components need special attention. For example, codeine (methylmorphine) is formulated as an antitussive agent in the form of a salt, such as codeine phosphate. In genetic polymorphisms of CYP2D6 (CYP2D6*2A, rs1080985), blood concentration of morphine, a metabolite of codeine, rises sharply and is secreted in breast milk. Deaths after infant breastfeeding have been reported when women with this genetic polymorphism took a high dose of codeine (e.g., 24 mg/day dihydrocodeine phosphate) as an analgesic [20]. Considering genetic polymorphisms, drugs containing codeine are not applied for children aged under 12 years in Japan [21], the E.U. [22], and the U.S. [23]. Although general nonprescription cold medicines marketed in Japan do not include a large amount of codeine, it should be noted that such unique constitutions exist. In addition, in terms of product design, nonprescription cold medicine often contains around 10 ingredients (e.g., codeine as an antitussive, diphenhydramine as an antihistamine, methylephedrine as a bronchodilator component). This large number of ingredients inhibits exact safety evaluations. Because cold medicines were frequently avoided by breastfeeding women in this study, it is recommended that simple and safe cold medicines are needed to support successful breast feeding and well-being.

In terms of oral analgesics, aspirin should be avoided because a previous study reported metabolic acidosis in infants fed breast milk from mothers taking a large amount of aspirin [5]. Furthermore, aspirin may be linked to Reye’s syndrome in infants [24]. However, acetaminophen, a first-line antipyretic analgesic for children, is marketed in Japan as a nonprescription drug suitable for breastfeeding women [19]. There have been only a few reports of potential adverse events in infants exposed to acetaminophen via breast milk [25,26]. The amount of acetaminophen transferred to milk by the mother’s intake is much less than the amount usually administered to infants [27,28,29].

Nonprescription drugs for menstrual pain and digestive medicine were also taken significantly less frequently in the BF group (0% and 1%, respectively) than in the FF group (13% and 13%, respectively) (*p* < 0.05) (Table 2). Because nonprescription drugs are medicines purchased based on symptoms and low-dose oral contraceptives are not available as nonprescription drugs in Japan, drugs for menstrual pain are most likely to be oral analgesics. However, the reason for significantly lower use of digestive medicines (*p* < 0.05) in the BF group is unclear, but may reflect breastfeeding women’s intention to reduce their intake of non-essential medicines. Prescription vitamins were significantly less used (*p* < 0.05) in the BF group (1%) than in the FF group (10%) (Table 3). This may also reflect the intention of health professionals to reduce non-essential medicines during breastfeeding or that of breastfeeding women to reduce their intake of non-essential medicines.

In this study, the percentages of consumption of nonprescription and prescription kampo medicine in the BF group (15% and 21%, respectively) were higher than in the FF group (10% and 3%, respectively). The BF group may have preferred to use kampo medicines because they considered these medicines more moderate and less toxic to infants than other medicines. This was consistent with a previous study from Italy, in which natural products were perceived as safer than drugs [30].

### 3.4. Sources of Drug Safety Information during Breastfeeding Period

Sources of drug safety information for breastfeeding women and the odds ratios for taking drugs were calculated between those who obtained information from a certain source and those who did not have this information (Table 4). For nonprescription drugs, “the Internet” was the most common information source (22%), followed by “pharmacist at the pharmacy medicine was purchased” (15%), “other doctor (e.g., family doctor)” (10%), “family/friends” (8%) and “other pharmacists” (7%). The odds ratios of taking medication when consulting “other doctors”, “pharmacist at the pharmacy medicine was purchased”, “other pharmacists”, or “family/friends” were 5.5, 5.1, 5.5, and 6.9, respectively. The rate of taking medication was significantly higher in those who received information from these sources than in those who did not receive information from these sources (Fisher’s exact test, *p* < 0.05). However, the group that received information from the Internet had the lowest odds ratio for taking medication. For prescription drugs, the most common information source was “prescribing doctor” (62%), followed by “the Internet” (29%), and “dispending pharmacist” (19%). The odds ratios for taking medication when consulting a doctor prescribing medicine or a dispensing pharmacist were 54 and 7.8, respectively (*p* < 0.05, Fisher’s exact test). However, the odds ratio for taking medication when the information was obtained from the Internet was only 1.8, with a nonsignificant difference. Although breastfeeding women commonly used the Internet to obtain information on prescription medicines, it did not appear to lead to medication use.

The result of those who used the Internet as a source of information had a lower odd ratio for taking medication suggested that information about taking medication available on the Internet while breastfeeding may be negative. The provision of drug information by health professionals was important for breastfeeding women, as there is information on the Internet about medicines for which the evidence is uncertain or not guaranteed. University education may be a key part of promoting the provision of drug information by health professionals. A previous survey of pharmacists working in community pharmacies in Nebraska in the U.S. reported that pharmacists who had worked for <30 years were considered more qualified to make nonprescription drug recommendations to lactating women than pharmacists with >31 years of experience [13]. Interestingly, this was consistent with the ratio of pharmacists that felt “the pharmacy school provided appropriate training regarding nonprescription drugs for lactating women”. This suggested that rather than experience (e.g., period of service years), it is formal pharmacy education that gives pharmacists confidence in providing drugs to breastfeeding women. However, few such studies have been conducted in Japan. Nonetheless, women in the present study received less information from pharmacists than other sources, such as the Internet. The opportunities for pharmacists to consult with mothers on their medication should be increased to prevent possible adverse effects, even for nonprescription drugs. Barriers to and gaps in such communication should also be investigated.

Only 25% of the participants did not ask/search for drug information for prescription drugs, whereas 44% of participants did not ask/search for nonprescription drug information. Those who stated “not asked, not searched” were those who did not need to take any medication, and therefore did not need to search for information, or they may have believed that nonprescription drugs were relatively safe compared with prescription medicines. Further studies are needed to analyze this issue in this population in more depth.

Because of the limited number of participants in this study, a stratified analysis by information source choice was not performed. However, a pilot correlation analysis suggested “the Internet”, “sales clerks at pharmacy of purchase”, and “drug package inserts” appeared to have been related to some extent (data not shown). It is possible that our sample was relatively self-reliant in seeking drug information. Interestingly, for both nonprescription and prescription drugs, women with more sources of information were more likely to take medication (Table 5). Because this was a cross-sectional study, it was unclear whether more sources of information led to higher medication use rates, or whether women with more sources of information were more likely to take medication if they needed to. Further research is needed to clarify these points.

### 3.5. Limitations of this Study

A major limitation of the study was that limited information was collected in the questionnaire, which has implications for the results presented and discussed in this paper. Therefore, this research should be considered an exploratory study. In further surveys, the questionnaire should collect information on aspects such as: (1) The quantity or regularity of the use of drugs, (2) Perceptions of risk and efficacy with comparison of non-prescribed and prescribed drugs as well as natural products, and (3) Trust in the source of the information. (1) The quantity or regularity of the use of drugs because the risk differs by whether the use of a drug is occasional or prolonged. (2) The failure to consider risk perception was another important limitation of this study. Information on breastfeeding women’s risk perceptions is crucial to understand their decisions to use/not use medicines during lactation [31]. It is also important to consider how breastfeeding women weigh efficacy versus risk when deciding to use medicines. (3) Further analyses should include trust in the source of the information as well as the source of the information as this information is also important for understanding decisions about drug use.

There were several other limitations in this study. Because this study used a cross-sectional design, it was difficult to control potential confounders between the compared groups and was not possible to clarify exact causal relationships. Secondly, it was possible that selection bias existed in terms of women who visited the childcare events at the community pharmacies. For example, the women targeted in this study may have had more time available than those who did not attend such events. Thirdly, the number of participants was relatively small, especially for the formula feeding only group. These differences may have affected the findings.

## 4. Conclusions

Our results suggested that breastfeeding plays an important role in women’s decisions about taking medication in Japan. Many breastfeeding women were hesitant about taking nonprescription drugs. This study also revealed that there are insufficient drug information sources in Japan. A major limitation of this study was the limited information that was collected with the questionnaire, which has implications for interpretation of the results. Qualitative studies could contribute to deepening knowledge on these issues. To promote healthy breastfeeding, it is important to provide sufficient drug safety information, and information on how to manage medicines during the breastfeeding period. Barriers and gaps in communication should also be investigated in a further study.

## Figures and Tables

**Table 1:** Relationships between medication use and breastfeeding.

			Breastfed Only (n = 46)	Breastfed More Than Half (n = 51)	Formula-Fed More Than Half (n = 20)	Formula Fed-Only (n = 11)	*p*-Value Among/Between the Groups	
			n (%)	n (%)	n (%)	n (%)	Cochran–Armitage Test for Trend	Fisher’s Exact Test
(Total, N = 128)								
Q1	Nonprescription drug use among women whose children were in infancy	Yes	12 (26)	21 (41)	11 (55)	9 (82)	**0.0003**	**0.004**
Q2	Prescription drug use among women whose children were in infancy	Yes	28 (61)	28 (55)	16 (80)	8 (73)	0.1705	0.2303
	*p*-value between the questions (Fisher’s exact test)		**0.002**	0.176	0.176	1.000		
(Formula feeding group, n = 31)								
Q3	Formula feeding because taking nonprescription drugs	Yes	n.a.	n.a.	0 (0)	0 (0)	n.a.	n.a.
Q4	Formula feeding because taking prescription drugs	Yes	n.a.	n.a.	0 (0)	1 (9)	n.a.	0.3548
	*p*-value between the questions (Fisher’s exact test)		n.a.	n.a.	n.a.	1.000	n.a.	
(Breastfeeding group, n = 97)								
Q5	Use of certain nonprescription drugs discontinued because of breastfeeding.	Yes	34 (74)	24 (47)	n.a.	n.a.	n.a.	**0.0079**
Q6	Use of certain prescription drugs discontinued because of breastfeeding.	Yes	20 (43)	20 (39)	n.a.	n.a.	n.a.	0.6853
	*p*-value between the questions (Fisher’s exact test)		**0.0056**	0.5489	n.a.	n.a.	n.a.	

Bold indicates significant difference (*p* < 0.05). n.a., not applicable.

**Table 2:** Women’s use of nonprescription drugs in the postpartum period.

Type of Drugs ^a^	Breastfeeding (BF) Group (n = 97)		Formula Feeding (FF) Group (n = 31)		*Difference*^b^ (BF–FF)	*p*-Value ^c^
	n	(%)	n	(%)	*%*	
**Cold medicines**	4	4	11	35	*−31*	**<0.001**
**Oral analgesics (medicines for headache and pain relief)**	7	7	8	26	*−19*	**0.009**
**Drugs for menstrual pain**	0	0	4	13	*−13*	**0.003**
**Digestive medicines**	1	1	4	13	*−12*	**0.012**
Drugs for skin blotches	0	0	2	6	*−6*	0.057
Drugs for hay fever	2	2	2	6	*−4*	0.247
Motion sickness drugs	0	0	1	3	*−3*	0.242
Eczema drugs	0	0	1	3	*−3*	0.242
Vitamin preparations	4	4	2	6	*−2*	0.632
Anti-itching cream for insect bites/insect repellents	1	1	1	3	*−2*	0.427
Vulnerary	1	1	0	0	*1*	1.000
Fomentation	2	2	0	0	*2*	1.000
Other supplements	5	5	0	0	*5*	0.335
Kampo medicine ^d^	15	15	3	10	*6*	0.559

^a^ Nonprescription drugs are medicines that are purchased at pharmacies based on symptoms, so some item descriptions for the options were different despite being the same type of medicines as for prescription drugs. Medicines not selected for any questions were athlete’s foot remedies, drugs for dry skin, acne medications, medical agents for constipation, drugs for muscle aches, drugs for stiff shoulders or back pain, drugs for healthy hair, and drugs for sleep. Bold font shows statistical significance (*p*-value < 0.05). ^b^ Percent point difference between drugs in the breastfeeding and formula-feeding groups. ^c^ Fisher’s exact test performed between drugs in the breastfeeding and formula-feeding groups. ^d^ Oriental medicines mainly based on herbal products used in Japan [18]. Bold indicates a significant difference (*p* < 0.05).

**Table 3:** Women’s use of prescription drugs in the postpartum period.

Type of Drugs ^a^	Breastfeeding (BF) Group (n = 97)		Formula Feeding (FF) Group (n = 31)		*Difference*^b^ (BF–FF)	*p*-Value ^c^
	n	(%)	n	(%)	*%*	
Cold medicines	23	24	12	39	*−15*	0.111
**Vitamins**	1	1	3	10	*−9*	**0.044**
Eczema drugs	2	2	3	10	*−8*	0.091
Antiallergic drugs	5	5	4	13	*−8*	0.219
Antiasthmatics	2	2	2	6	*−4*	0.247
Thyroid drugs	0	0	1	3	*−3*	0.242
Drugs for peptic ulcer/digestive medicines	5	5	2	6	*−1*	0.676
Analgesic ointments/creams	5	5	2	6	*−1*	0.676
Anti-infection drugs	12	12	4	13	*−1*	1.000
Drugs for postpartum hemorrhage	2	2	0	0	*2*	1.000
Antihypertensives	2	2	0	0	*2*	1.000
Laxatives	11	11	3	10	*2*	1.000
Iron preparations	8	8	1	3	*5*	0.687
Oral analgesics	31	32	8	26	*6*	0.655
**Kampo medicine ^d^**	20	21	1	3	*17*	**0.025**

^a^ Drugs not selected for any questions were antidepressive drugs, oral contraceptives, antithrombotic agents, anxiolytics, sedatives, antipsychotics, antirheumatics, antiacne preparations, blood sugar-lowering agents, lipid lowering agents, other cardiac drugs, antiepileptics, and ovulation inducing drugs. Bold font shows statistical significance (*p*-values <0.05). ^b^ Percent point difference between the breastfeeding and formula-feeding groups. ^c^ Fisher’s exact test performed between medicated drugs in the breastfeeding and formula-feeding groups. Bold indicates significant difference (*p* < 0.05). ^d^ Oriental medicines mainly based on herbal products used in Japan [18].

**Table 4:** Drug information sources for the breastfeeding group (n = 97), with multiple choice format.

	Nonprescription Drugs						Prescription Drugs					
	Info. Sources		Drug Use				Info. Sources		Drug Use			
Category of Information Source (Multiple Choice Format ^a^)	n	(%) ^d^	Yes	(%) ^e^	Odd Ratio ^b^	*p*-Value ^c^	n	(%) ^d^	Yes	(%) ^e^	Odd Ratio ^b^	*p*-Value ^c^
Prescribing doctor	n.a.	n.a.	n.a.	n.a.	n.a.	n.a.	60	62	52	87	54	**<0.001**
Other doctor (e.g., family doctor)	10	10	7	70	5.5	**0.03**	4	4	1	25	0.2	0.30
Dispensing pharmacist	n.a.	n.a.	n.a.	n.a.	n.a.	n.a.	18	19	16	89	7.8	**0.003**
Pharmacist at the pharmacy medicine was purchased	15	15	10	67	5.1	**0.01**	n.a.	n.a.	n.a.	n.a.	n.a.	n.a.
Other pharmacists	7	7	5	71	5.5	**0.04**	6	6	5	83	3.9	0.39
Sales clerks ^f^ at the pharmacy of purchase	2	2	2	100	n.a.	0.11	n.a.	n.a.	n.a.	n.a.	n.a.	n.a.
Nurses/midwives	4	4	3	75	6.3	0.11	6	6	5	83	3.9	0.39
Administrative agency (e.g., health center)	0	0	0	n.a.	n.a.	n.a.	0	0	0	n.a.	n.a.	n.a.
Family/friends	8	8	6	75	6.9	**0.02**	10	10	6	60	1.1	1.00
Books	1	1	0	0	n.a.	1.00	1	1	1	100	n.a.	1.00
Internet	21	22	8	38	1.3	0.80	28	29	19	68	1.8	0.25
Drug package inserts	6	6	4	67	4.2	0.17	n.a.	n.a.	n.a.	n.a.	n.a.	n.a.
Not asked, not searched (including no opportunity to take medication)	43	44	3	7	n.a.	n.a.	24	25	0	0	n.a.	n.a.

^a^ Participants selected information sources in multiple choice format, and the total number of sources exceeded 97. ^b^ Odds ratios for taking drugs were calculated between those who obtained information from a certain source and those who did not. ^c^ Statistical difference in taking medicine between those who obtained information from a certain source and those who did not (Fisher’s exact test). ^d^ Percentage in the breastfeeding group. ^e^ Percentage in each category. ^f^ Sales clerks licensed to sell nonprescription drugs. n.a., not applicable.

**Table 5:** Total number of drug information sources in the breastfeeding group (n = 97).

	Nonprescription Drugs				Prescription Drugs			
		Drug Use		Cochran–Armitage Test for Trend		Drug Use		Cochran–Armitage Test for Trend
Total Number of Information Sources (Total of Multiple Choice Items)	n	Yes	(%)	*p*-Value	n	Yes	(%)	*p*-Value
				<0.0001				<0.0001
0	43	3	7		24	0	0	
1	39	19	49		29	20	69	
2	8	6	75		32	26	81	
3	6	4	67		9	8	89	
4	1	1	100		2	1	50	
5	0	n.a.	n.a.		1	1	100	

n.a., not applicable.

## Supplementary Materials

The following are available online at https://www.mdpi.com/article/10.3390/ijerph191811722/s1, Table S1: Questionnaire on women’s medicine use and breastfeeding.
