# Supplementary material for: Use of Nonprescription and Prescription Drugs and Drug Information Sources among Breastfeeding Women in Japan: A Cross-Sectional Study
Source: Int J Environ Res Public Health. 2022 Sep 17;19(18):11722. doi: 10.3390/ijerph191811722 (PMC9517648; doi:10.3390/ijerph191811722)
Supplement: Supplementary file 1 [file ijerph-19-11722-s001.zip › ijerph-1833109-supplementary.pdf]

Supporting Information

Research Article

Use of non-prescription and prescription drugs and drug information sources among breastfeeding women in Japan: a cross-sectional study

Yukiko Fujii [1*], Keiko Hirokawa [2], Yuko Kobuke [1], Toshio Kubota [1], Taketo Yoshitake [1], Koichi Haraguchi [1], Yukiko Honda [3], Hatasu Kobayashi [4], Kouji H. Harada [5]

[1] Daiichi University of Pharmacy, Fukuoka 815-8511, Japan
[2] Sankyu-drug Ltd. Fukuoka 801-0825, Japan
[3] Department of Community Medicine, Nagasaki University Graduate School of Biomedical Sciences, Nagasaki 852-8523, Japan
[4] Department of Environmental and Molecular Medicine, Mie University Graduate School of Medicine, Mie 514-8507, Japan
[5] Department of Health and Environmental Sciences, Kyoto University Graduate School of Medicine, Kyoto 606-8501, Japan

[*] Correspondence: yu-fujii@umin.ac.jp

Table S1. Questionnaire on women's medicine use and breastfeeding

Information requested	Response
(All)	
Age of children (in months)	Written components
Parity	Written components
Type of feeding for childcare	Breastfeeding/formula feeding
Medicine use in the breastfeeding/formula feeding period	Yes/No; If "yes" was selected, the respondent was shown following lists of drugs<br><br>**Non-prescription drugs:** Cold medicines, medicines for headache and pain relief (oral analgesics), drugs for menstrual pain, digestive medicines, drugs for skin blotches, drugs for hay fever, motion sickness drugs, eczema drugs, vitamin preparations, anti-itching cream for insect bites/insect repellents, athlete's foot remedies, drugs for dry skin, acne medications, medical agents for constipation, drugs for muscle aches, drugs for stiff shoulders or back pain, drugs for healthy hair, drugs for sleep, vulnerary, fomentation, other supplements, kampo medicine<br><br>**Prescription drugs:** Cold medicines, vitamins, eczema drugs, antiallergic drugs, antiasthmatics, thyroid drugs, drugs for peptic ulcer/digestive medicine, analgesic ointments/creams, anti-infection drugs, antidepressive drugs, oral contraceptives, antithrombotic agents, anxiolytics, sedatives, antipsychotics, antirheumatics, antiacne preparations, blood sugar-lowering agents, lipid-lowering agents, other cardiac drugs, antiepileptics, ovulation inducing drugs, drugs for postpartum hemorrhage, antihypertensives, laxatives, iron preparations, oral analgesics, kampo medicine
(Formula feeding group only)	
I did not breastfeed because of taking non-prescription drugs	Yes/No
I did not breastfeed because of taking prescription drugs.	Yes/No
(Breastfeeding group only)	
I did not use a certain non-prescription drug because of breastfeeding	Yes/No
I did not use a certain prescription drug because of breastfeeding	Yes/No
Information sources for non-prescription and prescription drugs during the breastfeeding period	Respondent shown a list of sources (see Table 4)
